# Intrinsic supercurrent non-reciprocity coupled to the crystal structure of a van der Waals Josephson barrier

**DOI:** 10.1038/s41467-024-45298-9

**Published:** 2024-02-06

**Authors:** Jae-Keun Kim, Kun-Rok Jeon, Pranava K. Sivakumar, Jaechun Jeon, Chris Koerner, Georg Woltersdorf, Stuart S. P. Parkin

**Affiliations:** 1 Max Planck Institute of Microstructure Physics, Weinberg 2, 06120 Halle (Saale), Germany; 2 Department of Physics, Chung-Ang University (CAU), Seoul, 06974 Republic of Korea; 3 Department of Physics, Martin Luther University Halle-Wittenberg, Von-Danckelmann-Platz 3, 06120 Halle, Germany

**Keywords:** Superconducting properties and materials, Surfaces, interfaces and thin films

## Abstract

Non-reciprocal electronic transport in a spatially homogeneous system arises from the simultaneous breaking of inversion and time-reversal symmetries. Superconducting and Josephson diodes, a key ingredient for future non-dissipative quantum devices, have recently been realized. Only a few examples of a vertical superconducting diode effect have been reported and its mechanism, especially whether intrinsic or extrinsic, remains elusive. Here we demonstrate a substantial supercurrent non-reciprocity in a van der Waals vertical Josephson junction formed with a *T*_d_-WTe_2_ barrier and NbSe_2_ electrodes that clearly reflects the intrinsic crystal structure of *T*_d_-WTe_2_. The Josephson diode efficiency increases with the *T*_d_-WTe_2_ thickness up to critical thickness, and all junctions, irrespective of the barrier thickness, reveal magneto-chiral characteristics with respect to a mirror plane of *T*_d_-WTe_2_. Our results, together with the twist-angle-tuned magneto-chirality of a *T*_d_-WTe_2_ double-barrier junction, show that two-dimensional materials promise vertical Josephson diodes with high efficiency and tunability.

## Abstract (web summary)

J.-K. Kim et al. study vertical Josephson junctions where the weak link is *T*_d_-WTe_2_ and the superconductor is NbSe_2_. The use of an inversion-symmetry-breaking *T*_d_-WTe_2_ weak link allows the authors to demonstrate the intrinsic origin of the observed Josephson non-reciprocity in these devices.

## Introduction

The non-reciprocal behavior in dissipative current flows, known as the diode effect, has played a central role in modern electronic devices and circuits^1,2^. In conventional schemes, non-reciprocity along the current direction arises from spatial inhomogeneity^1,2^. Recently, it has been shown that when inversion and time-reversal symmetries are both broken, and in combination with a spin-orbit interaction (SOI), even spatially homogeneous systems can provide for diode functionality^3–5^. By implementing this magneto-chirality with superconductors (SCs) and matching the superconducting gap with the SOI energy^6,7^, one can achieve a directional, non-dissipative, supercurrent flow, which is a prerequisite for the realization of future superconducting quantum devices.

To date, several methods at the materials and device levels have been developed to intrinsically and/or extrinsically break the inversion symmetry. For example, via artificially patterned conformal arrays of nanoscale holes in a MoGe film^8^, via ionic-liquid gating of two-dimensional (2D) MoS_2_ flakes, and via the formation of non-centrosymmetric V/Nb/Ta superlattices or using a few layers of 2H-NbSe_2_ have enabled the observation of non-reciprocal critical currents near their corresponding superconducting transition temperatures *T*_c_^9–11^. Notably, non-reciprocity in Josephson supercurrents have also been realized in lateral Josephson junctions (JJs) with structures: Al/InAs 2D electron gas (2DEG)/Al^7^, Nb/proximity-magnetized Pt/Nb^12^, Nb/NiTe_2_ type-II Dirac semimetal/Nb^13^, three-terminal Al/InAs 2DEG/Al JJs^14^. To break the inversion symmetry, the first two utilize a Rashba(-type) superlattice and barrier, respectively, the third one exploits topological surface states in a NiTe_2_ barrier, whereas the last one employs the geometric breaking of the device’s inversion symmetry by the presence of third terminal. On the other hand, there have been only a few relevant studies on vertical JJs. A field-free Josephson diode effect has been recently observed in vertical NbSe_2_/Nb_3_Br_8_/NbSe_2_ junctions^15^, but the non-reciprocal supercurrents appear in the direction along which the inversion symmetry is broken and the diode polarity is, moreover, independent of an applied magnetic field, making investigations of other devices highly desirable to unravel the role of van der Waals barriers. Moreover, whether or not there can be an intrinsic Josephson diode effect in such vertical junctions is the outstanding quest in this increasing research field of supercurrent non-reciprocity.

Very recent studies^16,17^ on the Al/InAs 2DEG/Al lateral junctions have investigated a possible correlation between the bulk inversion symmetry breaking (i.e. Dresselhaus-type) and the supercurrent non-reciprocity. In the case of interface inversion symmetry breaking (i.e. Rashba-type), the associated spin-orbit (SO) field in *k*-space is fundamentally isotropic^16^. When the Josephson barrier possesses the Dresselhaus-type symmetry breaking in addition to the Rashba-type symmetry breaking, the overall SO field becomes anisotropic, making the magneto-chiral anisotropy of supercurrent non-reciprocity dependent of the Josephson barrier’s crystal structure^16^. However, the strength of Dresselhaus-type SO field in such lateral devices turns out to be quite small (<10% at most) as compared with that of Rashba SO field. For this reason, to the best of our knowledge, the lateral device geometry is less suited for the investigation of supercurrent non-reciprocity derived solely from the bulk inversion symmetry breaking of the Josephson barrier.

Here, we utilize *T*_d_-WTe_2_ single-crystal exfoliated flakes as an inherently inversion symmetry breaking barrier in van der Waals (vdW) JJs and through extensive measurements of the *T*_d_-WTe_2_ flake thickness, magnetic field strength/angle and temperature dependences, we demonstrate that the supercurrent non-reciprocity along the vertical direction is intimately connected and is highly dependent on the crystal structure of the *T*_d_-WTe_2_ barrier, thereby allowing a clear distinction between intrinsic and extrinsic mechanisms^7,12,13^ and providing the experimental realization of the intrinsic JDE that results *purely* from the bulk inversion symmetry breaking of the *T*_d_-WTe_2_ barrier. Our study establishes that the *co-existence* of the magneto-linearity, the magneto-chirality and the thickness and temperature-scaleable diode efficiency constitute the *intrinsic* supercurrent non-reciprocity, coupled to the crystal structure of a vdW barrier.

## Results

**Fig. 1:**
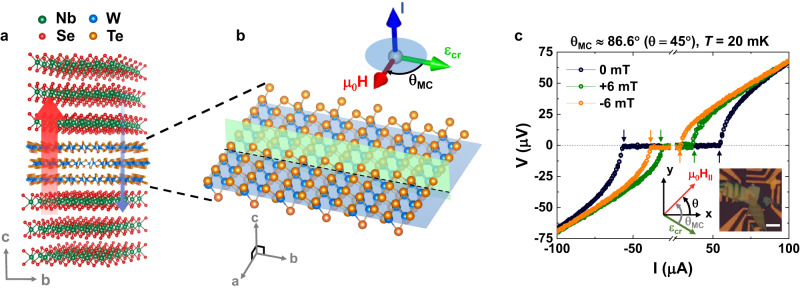
Vertical rectified supercurrents in a van der Waals WTe_2_ Josephson junction. **a** Schematic of a NbSe_2_/*T*_d_-WTe_2_/NbSe_2_ van der Waals vertical Josephson junction (JJ). The combination of intrinsic inversion symmetry breaking within the *T*_d_-WTe_2_ barrier and time-reversal symmetry breaking by an applied magnetic field give rise to the (vertical) supercurrent non-reciprocity. **b** Schematic diagram of the crystal structure of WTe_2_. Orange (blue) symbols represent the W (Te) atoms. *θ*_*MC*_ is defined as the relative angle between the polar axis along *b*-axis, the mirror plane of *T*_d_-WTe_2_ (green plane), and the direction of the in-plane (IP) magnetic field ${\mu }_{0}{H}_{\parallel }$ applied within the *a*-*b* plane of *T*_d_-WTe_2_ (blue plane). **c** Current-voltage *I–V* curves of a van der Waals JJ with a 23 nm thick *T*_d_-WTe_2_ barrier for ${\mu }_{0}{H}_{\parallel }$ = 0 (black), +6 (orange) and −6 (green) mT. Note that $\theta$ is the angle of ${\mu }_{0}{H}_{\parallel }$ relative to an edge of the rectangular Si wafer on which the JJ was formed.

As illustrated in Fig. 1a, we fabricate vdW vertical JJs, in which a *T*_d_-WTe_2_ flake (2−60 nm thick) is sandwiched between two superconducting 2*H*-NbSe_2_ flakes, in an inert atmosphere glovebox using dry transfer techniques (see Methods for details). Here, we employed the NbSe_2_ flakes thicker than 10 nm (16−17 $\times$ the monolayer thickness) to preclude the unintended contribution of Ising Cooper paring^18^ to the non-reciprocal transport properties of WTe_2_ JJs. Note that *T*_d_-WTe_2_ itself exhibits highly interesting physical properties including a type-II Weyl semi-metallic behavior^19^, higher-order topological hinge states^20^ and quantum spin-Hall edge states^21^. In the present study, we focus on how the crystal inversion symmetry of *T*_d_-WTe_2_ is reflected in a vertical Josephson diode effect. Due to the lack of a screw rotational symmetry in *T*_d_-WTe_2_ from the Te atoms, the orthorhombic phase *T*_d_-WTe_2_ is non-centrosymmetric^20,22,23^. The mirror-symmetry plane of *T*_d_-WTe_2_ along the *b*-axis (green plane in Fig. 1b) creates a polar axis, that is, an internal crystal electric field ${\varepsilon }_{{cr}}$^22^ and together with the heavy W atoms provide a strong SOI. This, in conjunction with an external in-plane (IP) magnetic field ${\mu }_{0}{H}_{\parallel }$, that breaks the time reversal symmetry, allows for an anomalous phase ${\varphi }_{0}$ to be created in the current-phase relationship (CPR) of the vertical JJ^24–26^. As previously discussed theoretically^24–26^, the presence of a finite ${\varphi }_{0}$ is essential to generate a rectified Josephson supercurrent, namely, unequal positive and negative Josephson switching supercurrents ${I}_{c}^{+} \, \ne \, {I}_{c}^{-}$^7,12^.

Below, we will separate a crystal-asymmetry-driven intrinsic diode effect from other extrinsic possibilities in the following manner. When the dc bias current *I* is applied along the vertical direction (// *c*-axis) and the IP magnetic field is applied in the *a-b* plane of the WTe_2_ (blue plane in Fig. 1b), the Josephson diode efficiency $\eta=\frac{{I}_{c}^{+}-|{I}_{c}^{-}|}{{I}_{c}^{+}+|{I}_{c}^{-}|}$ is given by ${\eta \propto \varphi }_{0}\propto {\mu }_{0}{H}_{\parallel }\cdot \sin ({\theta }_{{MC}})$. Here ${\theta }_{{MC}}$ is the relative angle between the polar axis (// *b*-axis) of WTe_2_ and the applied ${\mu }_{0}{H}_{\parallel }$^7,24–26^. The magneto-linearity ($\propto {\mu }_{0}{H}_{\parallel }$) and the magneto-chirality [$\propto \sin ({\theta }_{{MC}})$] of $\eta$ are key measures of the intrinsic Josephson diode effect.

Figure 1c displays representative current-voltage (*I–V)* curves for a NbSe_2_/*T*_d_-WTe_2_/NbSe_2_ vdW JJ formed with a 23-nm-thick *T*_d_-WTe_2_ barrier. *I–V* curves are shown when ${\mu }_{0}{H}_{\parallel }$ = 0 (black), +6 (orange) and −6 (green) mT are applied at a fixed ${\theta }_{{MC}}$ ≈ 86.6^o^ ($\theta$ = 45^o^). These measurements are performed at 20 mK, far below the junction’s *T*_c_ $\approx$ 5 K. $\theta$ is the angle of ${\mu }_{0}{H}_{\parallel }$ relative to an edge of the Si wafer on which the exfoliated layers were placed: the wafer had been cut into a rectangular shape to define a clear reference direction. Note that ${\theta }_{{MC}}$ in Fig. 1c is determined from comparison of the ${\mu }_{0}{H}_{\parallel }$ angular dependence of $\eta$ of JJ with polarization-resolved Raman spectra (see Supplementary Information S3 for details). It is clear that a critical current asymmetry ($\triangle {I}_{c}={I}_{c}^{+}-\left|{I}_{c}^{-}\right|\ne 0$) is only developed when ${\mu }_{0}{H}_{\parallel }$ is non-zero and that the polarity of $\triangle {I}_{c}$ is inverted when the direction of ${\mu }_{0}{H}_{\parallel }$ is reversed. These features correspond to a Josephson diode effect. Note that since our *T*_d_-WTe_2_ barrier JJs show a weak hysteresis in the *I–V* curves, indicating overdamped characteristics and the ignorable re-trapping, these can thus be the best example of experimental investigating the intrinsic Cooper-pair tunneling non-reciprocity by circumventing the unintentional contribution of quasiparticle tunneling^25^ to the non-reciprocity.

**Fig. 2:**
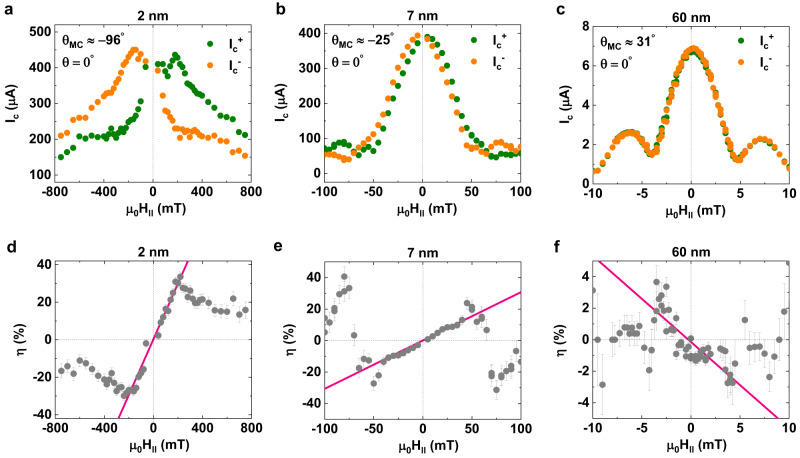
Scaling of Josephson supercurrent non-reciprocity with magnetic field strength. **a**–**c** Positive and negative Josephson critical current ${I}_{c}^{+}$ (green) and $\left|{I}_{c}^{-}\right|$ (orange) *versus* in-plane (IP) magnetic field ${\mu }_{0}{H}_{\parallel }$ for **a** 2 nm, **b** 7 nm, and **c** 60 nm thick *T*_d_-WTe_2_ barriers in a NbSe_2_/*T*_d_-WTe_2_/NbSe_2_ Josephson junction. The measurement was conducted at *T* = 200 mK for the 2 nm and 7 nm thick junctions and *T* = 20 mK for the 60 nm thick junction, respectively. Note that the *I–V* curves of 7 nm thick barrier JJs reveal no significant difference between 200 and 20 mK (Supplementary Information S7), as would be expected for the measurement temperature of ≤ 0.3 *T*/*T*_c_ (Fig. 4b). **d**–**f** Josephson diode efficiency ($\eta=\frac{{I}_{c}^{+}-\left|{I}_{c}^{-}\right|}{{I}_{c}^{+}+\left|{I}_{c}^{-}\right|}$) as a function of ${\mu }_{0}{H}_{\parallel }$ for **d** 2 nm, **e** 7 nm, and **f** 60 nm thick *T*_d_-WTe_2_ barriers. Note that the pink fits indicate a linear scaling behavior of the diode efficiency with ${\mu }_{0}{H}_{\parallel }$ up to a critical field, above which the diode efficiency drops.

The magneto-linearity of $\eta (\propto {\mu }_{0}{H}_{\parallel })$ is first checked by measuring how ${I}_{c}^{+}$ and $\left|{I}_{c}^{-}\right|$ vary with the strength of ${\mu }_{0}{H}_{\parallel }$ for a fixed ${\theta }_{{MC}}\ne$ 0^o^ ($\theta$ = 0^o^). From a comparison of$\,\left|{I}_{c}({\mu }_{0}{H}_{\parallel })\right|$ and $\eta ({\mu }_{0}{H}_{\parallel })$ for several values of the thickness of WTe_2_ (2, 7, and 60 nm) (Fig. 2a−f), three notable features are revealed. First, for all the JJs, $\eta$ increases linearly with increasing ${\mu }_{0}{H}_{\parallel }$ up to a certain critical field, above which $\eta$ starts to decay (Fig. 2d−f). Second, when this field coincides with the first-order minimum field of the Fraunhofer interference pattern, $\eta$ decays in a complex way with an accompanying sign change (Fig. 2b, c, e, and f). Third, the slope of the low-field $\eta ({\mu }_{0}{H}_{\parallel })$ data (Fig. 2d−f) depends critically on the orientation of the polar axis of the WTe_2_ with respect to the applied ${\mu }_{0}{H}_{\parallel }$. While the first and second features are qualitatively similar to previous studies on Al/2DEG/Al^7^ and Nb/NiTe_2_/Nb^13^ lateral JJs, the third, signifying the magneto-chirality of $\eta [\propto \sin ({\theta }_{{MC}})]$, is a new finding from the NbSe_2_/WTe_2_/NbSe_2_ vertical JJs. Note that no signature of fast oscillations, often attributed to topological edge states of WTe_2_^27^, are detected in measurements of the magnetic-field interference patterns for our vertical JJs (Fig. 2a−c). This is in agreement with theoretical considerations that topological edge states, that reside on the *a-b* planes of WTe_2_^28^_,_ may contribute to lateral transport^29,30^ but not to vertical transport. This indicates the crystallographic origin of the WTe_2_ barrier for the magneto-chirality that we report here.

**Fig. 3:**
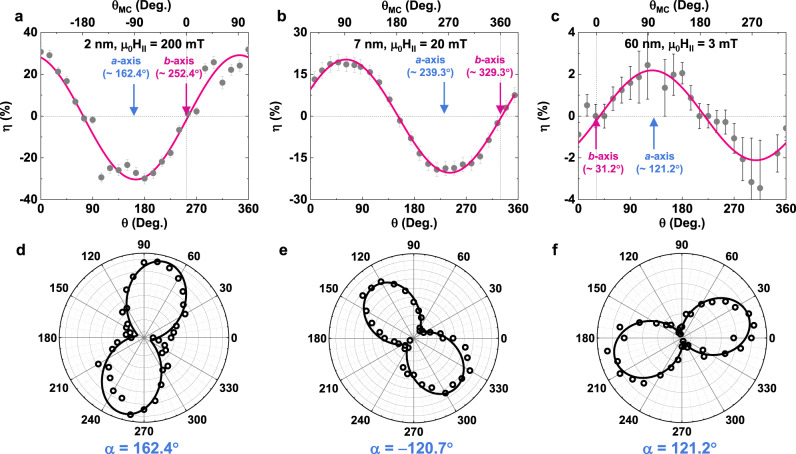
Magneto-chiral angular dependence of the supercurrent non-reciprocity on the WTe_2_ crystal structure. **a**–**c** Field angle dependent Josephson diode efficiency ($\eta=\frac{{I}_{c}^{+}-|{I}_{c}^{-}|}{{I}_{c}^{+}+|{I}_{c}^{-}|}$) of NbSe_2_/*T*_d_-WTe_2_/NbSe_2_ junctions with **a** 2 nm, **b** 7 nm and **c** 60 nm thick *T*_d_-WTe_2_ barriers. Pink lines represent sine fits to the data. Top *x*-axis of the graphs show the ${\theta }_{{MC}}$ of each WTe_2_ junctions. **d**–**f** Polarization angle dependent relative intensity of two distinct Raman peaks ( ~ 160 cm^−1^ and ~_2_10 cm^-1^), from which the *a*- and *b*-axis of the WTe_2_ can be determined. The *a*-axis and *b*-axis directions of the WTe_2_ flake found in this way are indicated by arrows in (**a**–**c**).

To confirm this distinctive magneto-chirality, the ${\mu }_{0}{H}_{\parallel }$ angular dependence of $\eta$ is measured for each JJ. Note that this measurement is conducted in the low-field regime where the magneto-linearity of $\eta (\propto {\mu }_{0}{H}_{\parallel })$ holds (see Fig. 2d−f). As summarized in Fig. 3a−c, the measured $\eta (\theta )$ data are all well fitted by a sine function, suggesting a magneto-chiral origin of the Josephson diode effect in the vdW WTe_2_ JJs. Furthermore, there exists a visible shift between the sine fits to the $\eta (\theta )$ data for different WTe_2_ JJs. This indicates that the crystal structure of the WTe_2_ barrier indeed governs the Josephson supercurrent non-reciprocity in our system. The magneto-chirality described above predicts $\eta$ to be maximized (minimized) when the applied ${\mu }_{0}{H}_{\parallel }$ is aligned along the *a*-axis (*b*-axis). Note that in Fig. 3a−c, there exist non-zero constant offsets that are irrelevant to the intrinsic supercurrent rectification of magneto-chiral origin^12^. It is highly likely that such constant offsets arise from the asymmetric interface of the junctions as discussed previously^15^. Furthermore, a small out-of-plane component of the applied magnetic field due to a misalignment can, in principle, generate the field-angle-independent offset (Supplementary Information S9). To identify the *a-* and *b*-axis of each WTe_2_ barrier in the fabricated junctions, angle-resolved polarized Raman spectroscopy was carried out: this technique can readily characterize the crystal orientation of low-dimensional materials^20,22^. Figure 3d−f, show the polarization angle dependent relative intensities of two distinct Raman peaks at ~160 cm^-1^ and ~210 cm^-1^. The extracted *a*- and *b*-axis of the WTe_2_ from the Raman data (Fig. 3d−f, see Supplementary Information S3 for the detailed analysis) agree with those from the $\eta (\theta )$ data (Fig. 3a−c). These results strongly support an intrinsic-crystal-structure-reflected vertical Josephson diode effect. For completeness, we also fabricated and measured control vdW JJs, where the non-centrosymmetric WTe_2_ barrier is replaced with a MoTe_2_ barrier that is locally centrosymmetric and may have a mixed *T*_d_/1 *T*′ structure (see Supplementary Information S4)^31^. In addition, the effect of direct tunneling between the top and bottom superconducting NbSe_2_ electrodes in JJs without any vdW-WTe_2_ (Supplementary Information S6) were fabricated. None of the magneto-linearity and the magneto-chirality effects are found in these experiments (see Supplementary Information S4), thereby pinpointing the critical role of the Josephson barrier’s crystal structure for the supercurrent non-reciprocity in our WTe_2_ JJs. Based on these results, we can rule out other extrinsic possibilities for the supercurrent non-reciprocity in our WTe_2_ JJs, e.g., pinning of Abrikosov vortices or a kinetic inductance contribution which have little to do with the crystal structure of the Josephson barrier (see Supplementary Information S8 for more discussions). In addition, one may expect the vdW gap formed at either side of the WTe_2_ Josephson barrier to function as a tunnel barrier at the interface between NbSe_2_ and WTe_2_ flakes. As shown by the exponential decay of the characteristic voltage of WTe_2_ JJs as a function of the thickness of the WTe_2_ barrier (Supplementary Information S5), this is the case and so, our WTe_2_ JJs need to be regarded as superconductor-insulator (vdW gap)-normal metal-insulator-superconductor (SINIS) junctions. This is in contrast with a previous study on lateral Nb/WTe_2_/Nb JJs^32^ that in the case of ballistic junctions with transparent/direct contacts, the barrier-thickness-dependent characteristic voltage is expected to reveal the 1/*L* dependence in the long junction limit. Here *L* is the lateral spacing of the neighboring Nb electrodes. Nevertheless, given the presence (absence) of crystal-structure-coupled magneto-chirality in WTe_2_ (MoTe_2_) JJs, it is unlikely that the crystal-structure-less vdW gaps play a significant role in producing such a characteristic magneto-chirality.

**Fig. 4:**
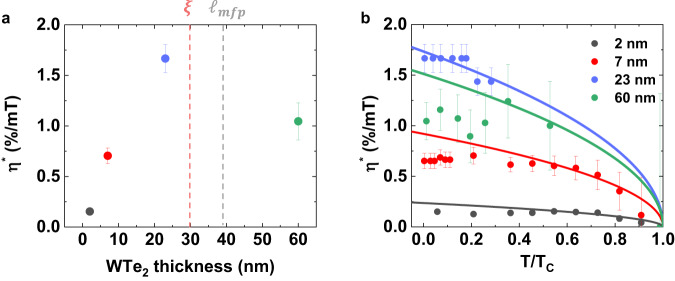
Thickness and temperature dependent Josephson supercurrent non-reciprocity. **a** Field-normalized Josephson diode efficiency ${\eta }^{*}$ ($=\frac{{I}_{c}^{+}-\left|{I}_{c}^{-}\right|}{{I}_{c}^{+}+\left|{I}_{c}^{-}\right|}/{\mu }_{0}{H}_{{||}}$, measured at *θ*_MC_ = 90°) as a function of the *T*_d_-WTe_2_ barrier thickness. Vertical red and gray dashed lines correspond to the coherence length (Supplementary Information S4) and *c*-axis mean free path^22^ of the *T*_d_-WTe_2_. **b** Normalized dependence of ${\eta }^{*}$ as a function of normalized temperature *T/T*_*c*_ of NbSe_2_/*T*_d_-WTe_2_/NbSe_2_ Josephson junctions with different *T*_d_-WTe_2_ barrier thicknesses (2, 7, 23 and 60 nm). The *T*_*c*_ values of WTe_2_ JJs are 5.5 K (2 nm and 7 nm), 5 K (23 nm) and 1.5 K (60 nm), respectively. Solid lines represent fits to the data of $\sqrt{1-\frac{T}{{T}_{c}}}$.

The proportionality of ${\varphi }_{0}$ to the barrier length is another characteristic feature of the intrinsic Josephson diode effect^24–26^, so one can anticipate that the thicker the WTe_2_ barrier, the greater the diode efficiency. To test this, we next investigate how the field-strength-normalized diode efficiency ${\eta }^{*}=\frac{\eta }{{\mu }_{0}{H}_{\parallel }}$, measured at *θ*_MC_ = 90°, scales with the WTe_2_ thickness (Fig. 4a). Note that since $\eta$ $\propto$ ${\mu }_{0}{H}_{\parallel }$ in the low-field regime, ${\eta }^{*}$ ($=\frac{\eta }{{\mu }_{0}{H}_{\parallel }}$) allows for a more quantitative comparison. As the WTe_2_ thickness is increased from 2 to 23 nm, we find that ${\eta }^{*}$ increases linearly. This linear scaling behavior is, in fact, predicted theoretically for a ballistic JJ^24^. When the thickness of the WTe_2_ is increased to 60 nm, which is larger than the coherence length (~30 nm, Supplementary Information S5) and the *c*-axis mean free path (~40 nm)^33^, ${\eta }^{*}$ deviates from the linear thickness dependence. Given the distinctively different barrier-thickness dependence of ${\varphi }_{0}$ on whether the junction is in the ballistic or diffusive regime, such a deviation is likely related to a ballistic-to-diffusive (long-junction) transition^24–26^. As the *c*-axis mean free path is close to the *c*-axis coherence length of our WTe_2_ JJs, one should also consider a short-to-long junction transition^32^ *within* the ballistic limit (Supplementary Information S5). To clarify whether the non-monotonic thickness-dependent ${\eta }^{*}$ results from the ballistic-to-diffusive transition^24–26^ or the short-to-long ballistic transition^32^, further study is necessary in the future.

The temperature *T* dependent evolution of η*, gives additional information about the intrinsic Josephson diode properties. Figure 4b shows the *T/T*_*c*_-dependent η*for several WTe_2_ JJs. It is especially noteworthy that the ${\eta }^{*}(T/{T}_{c})$ data can be well described by a $\scriptstyle \sqrt{1-\frac{T}{{T}_{c}}}$ function for$\,\frac{T}{{T}_{c}}\gtrsim \frac{1}{3}$, as predicted in several earlier theoretical studies^24–26,34–36^. For$\,\frac{T}{{T}_{c}} < \frac{1}{3}$, ${\eta }^{*}$ tends to saturate, which is qualitatively similar to recent related experiments^7,11^. A very recent theory predicts a rather complicated *T*-dependence of the superconducting diode effect in diffusive Rashba-type SCs^35^ as the low-*T* limit diode efficiency is affected sensitively by structural disorder and impurity scattering. On the other hand, for Al/2DEG/Al ballistic JJs^7^, the field-strength-dependent supercurrent non-reciprocity is explained by considering contributions from first and second order harmonics in the JJ CPR. If we apply this analysis to our ${\eta }^{*}(T/{T}_{c})$ data, both harmonics seem to become constant at $\frac{T}{{T}_{c}} < \frac{1}{3}$, as is consistent with the calculation in Ref. ^7^

**Fig. 5:**
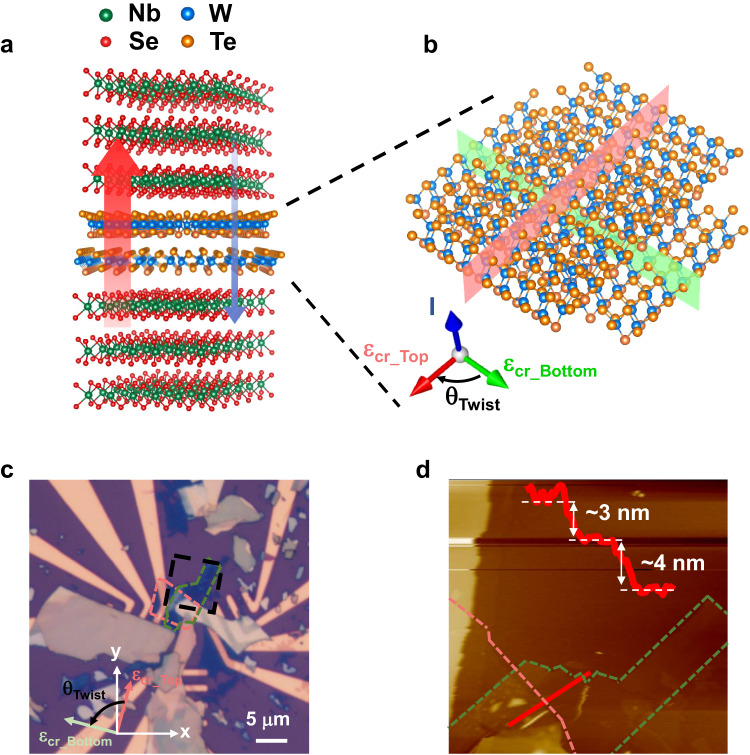
Fabrication of twisted WTe_2_ double-barrier JJ. **a** Schematic diagram of a NbSe_2_/twisted-WTe_2_ double-barrier/NbSe_2_ vdW vertical JJ. **b** magnified schematic of the crystal structure of the twisted-WTe_2_ double-barrier. Notably, the vector addition of the internal crystal fields ε_cr_Top_ and ε_cr_Bottom_ of the top and bottom WTe_2_ layers, respectively, with the twist angel *θ*_twist_ leads to a new artificial polar axis of the entire Josephson barrier. **c** Optical micrograph of the twisted-WTe_2_ double-barrier JJ. The top (bottom) WTe_2_ flake is marked by the red (green) dashed line. ε_cr_Top_ (ε_cr_Bottom_) is defined to be along the polar axis (*b*-axis) of the top (bottom) WTe_2_. These axes are directly probed by angle-resolved polarized Raman spectroscopy, as depicted on the optical micrograph. The black dashed box represents the area scanned by AFM as shown in (**d**). **d** AFM image of respective WTe_2_ barriers. Inset: depth profile along the red solid line.

**Fig. 6:**
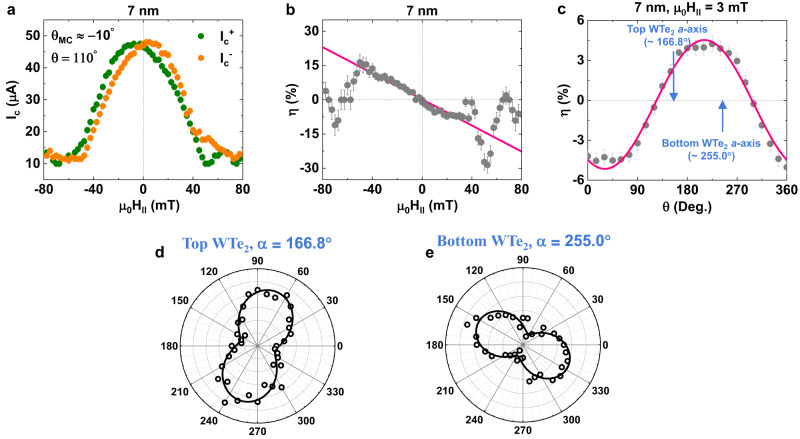
Tuned magneto-chirality using the artificial polar axis of a twisted WTe_2_ double-barrier JJ. **a** Positive and negative Josephson critical current ${I}_{c}^{+}$ (green) and $\left|{I}_{c}^{-}\right|$ (orange) *versus* in-plane (IP) magnetic field ${\mu }_{0}{H}_{\parallel }$ for twisted-WTe_2_ double barrier JJ. Josephson diode efficiency $\eta (=\frac{{I}_{c}^{+}-|{I}_{c}^{-}|}{{I}_{c}^{+}+|{I}_{c}^{-}|})$ as a function of magnetic field strength (**b**) and angle (**c**) for the twisted WTe_2_ double-barrier JJ. Polarization-angle-dependent relative intensity of two distinct R*a*man peaks (~160 cm^-1^ and ~210 cm^-1^), from which one can determine the *a*- and *b*-axes of the top (**d**) and bottom (**e**) WTe_2_ barriers. The measured *a*-axis dire**c**tions of the top and bottom WTe_2_ vdW layers are indicated by arrows in (**c**). As can be seen in (**c**) the magneto-chiral non-reciprocal supercurrents of the twisted-WTe_2_ double barrier JJ are successfully controlled by twisting the top WTe_2_ barrier relative to the bottom one.

To further show the importance of our vertical JJ platform, that goes beyond recent studies^7–12^, we have fabricated vertical JJs with a twisted WTe_2_ double-barrier (Methods). In these twisted double-barrier junctions, *the vector addition of the internal crystal fields of the top and bottom WTe*_*2*_ *layers*, respectively, with a twist angle between two layers leads to a new artificial polar axis of the entire Josephson barrier. Note that the twist angle ${\theta }_{{twist}}$ between two distinct WTe_2_ layers uniquely forms an artificial polar axis of the entire Josephson barrier in a controllable manner (Fig. 5). In this sense, one can expect that the ${\theta }_{{MC}}$ is determined with respect to this new artificial polar axis. As shown in Fig. 6c, the diode efficiency of a twisted WTe_2_ double-barrier JJ shows a visible sinusoidal behavior and the diode efficiency of the JJ goes to zero when the magnetic field is applied to the parallel direction of the vector addition of the internal crystal field of top and bottom WTe_2_ layers. This indicates that the magneto-chirality of twisted-double barrier JJ can be determined by the artificial polar axis of the barrier_,_ demonstrating a tunable magneto-chirality via twist-angle engineering and opens an avenue for the development of twistable^36^ active Josephson diodes.

We have carefully fabricated vdW vertical JJs with an inherently inversion symmetry breaking *T*_d_-WTe_2_ barrier. This has allowed us to unambiguously demonstrate the intrinsic origin of the Josephson non-reciprocity that we observe in these vertical JJs. We clearly demonstrate that the crystal structure of the *T*_d_-WTe_2_ Josephson barrier governs the overall properties of the Josephson diode effect, which has not been previously been shown. We have also shown that whether or not the magneto-linearity, the magneto-chirality and the thickness and temperature-scaleable diode efficiency coexist is a key criterion in distinguishing intrinsic and extrinsic mechanisms. We believe that our results have definitely shown the critical role of the barrier in the realization of rectified Josephson supercurrents in vdW heterostructures^15^ and provides the first demonstration of twist engineering of the magneto-chirality. Our approach can be extended to other low-dimensional, low-symmetric and twisted vdW systems^37–39^ for accelerating the development of two-dimensional superconducting devices and circuits.

## Methods

### Device fabrication

The NbSe_2_/WTe_2_/NbSe_2_ van der Waals (vdW) heterostructures (Fig. S1a−d) were formed using standard dry transfer techniques. All the needed processes, including mechanical exfoliation, pick-up and dry transfer of the vdW flakes, were performed inside a nitrogen gas filled glovebox to prevent the flakes from oxidizing in ambient air. First, NbSe_2_ ($\ge$ 10 nm thick) and WTe_2_ (2−60 nm thick) flakes were exfoliated on to a 300-nm-thick SiO_2_/p + + Si substrate. Each of the flakes needed to form the heterostructure was chosen by optical microscopy examination. The chosen flakes were picked up sequentially using a polycarbonate (PC) film coated dome-shaped polydimethylsiloxane (PDMS) stamp. The flakes were aligned one on top of each other and them the entire stack was released and then placed on top of a set of pre-patterned gold electrodes on a second 300-nm-thick SiO_2_/p + + Si substrate. The release process was performed at 200 C°, after which the entire stack in the Si substrate was immersed in a chloroform solution to remove any PC residue. Using this fabrication process flow, we also prepared three distinct types of JJs: (1) a control vdW JJ, in which the non-centrosymmetric WTe_2_ barrier^20,22,23^ is replaced by a MoTe_2_ barrier^31^ that may have a *mixed T*_d_/1 *T*′ structure and is to a certain extent locally centrosymmetric at low temperatures (Supplementary Information S4), (2) a reference vdW JJ with *no* WTe_2_ barrier (Supplementary Information S6), and (3) a twisted WTe_2_ double-barrier JJ (Figs. 5–6), where one WTe_2_ is twisted in-plane relative to the other WTe_2_. For the formation of the twisted WTe_2_ double-barriers, we first identified the crystal orientation of two chosen WTe_2_ flakes through their elongated shapes^20^ (which tends to be along the *a*-axis), and then carefully twisted and stacked one on top of the other to realize ${\theta }_{{twist}}$ ≈ 90°. After completing the transport measurements, we finally confirmed the respective crystal orientations of the top and bottom WTe_2_ flakes by a means of angle-resolved polarized Raman spectroscopy.

### Electrical measurements

The current-voltage (*I–V)* curves of the fabricated NbSe_2_/WTe_2_/NbSe_2_ vdW vertical Josephson junctions (JJs) were measured in a BlueFors dilution refrigerator or a Quantum Design Physical Property Measurement System with base temperatures of ~20 mK and ~1.8 K, respectively. The four-point *I*–*V* measurements were carried out using a Keithley 6221 current source and a Keithley 2182 A nanovoltmeter. We determined the critical current *I*_*c*_ values at the point where *V(I)* ≈ 1 μV. An external in-plane magnetic field (${\mu }_{0}{H}_{\parallel }$), that was needed to break the time-reversal symmetry, was applied within the *a-b* plane of the WTe_2_ single crystalline barrier. Note that $\theta$ is the angle of ${\mu }_{0}{H}_{\parallel }$ relative to one edge of the Si wafer on which the exfoliated layer stack was placed: the wafer had been purposely cut into a rectangular shape to define a clear reference direction. The typical Josephson penetration depth^40^ of our junctions (of at least a few μm) is much larger than the WTe_2_ thickness, so we can exclude orbital magnetic field effects (or Meissner demagnetizing supercurrents) as a possible extrinsic source for of the Josephson diode effect in our JJs. We measured the field strength dependent diode signals (Fig. 2a−c) by applying ${\mu }_{0}{H}_{\parallel }$ along a given $\theta$ direction. The angle dependent diode signals (Fig. 3a–c) were measured at a constant ${\mu }_{0}{H}_{\parallel }$ using a vector field magnet.

### Atomic force microscopy

To characterize the thickness of each WTe_2_ barrier, we conducted atomic force microscopy (AFM) measurements on the fabricated vdW vertical JJs. The measured AFM image and height of each WTe_2_ barrier layer are shown together with the corresponding optical micrograph of the JJs in Fig. S1.

### Supplementary information

Supplementary Information

Peer Review File

## Data Availability

The data that support the findings of this study are provided in main text and the Supplementary Information. The original data available from the corresponding authors upon request.

## References

[1] Sze, S. M. & Kwok, K. N. in *Physics of Semiconductor Devices* 793–815 (John Wiley & Sons, 2006).

[2] Neamen, D. A. *Semiconductor Physics and Devices: Basic Principles*. Vol. 528 (McGraw-Hill, 2012).

[3] Onsager L (1931). Reciprocal relations in irreversible processes. I. Phys. Rev..

[4] Kubo R (1957). Statistical-mechanical theory of irreversible processes. I. General theory and simple applications to magnetic and conduction problems. J. Phys. Soc. Jpn..

[5] Rikken GLJA, Fölling J, Wyder P (2001). Electrical magnetochiral anisotropy. Phys. Rev. Lett..

[6] Tokura Y, Nagaosa N (2018). Nonreciprocal responses from non-centrosymmetric quantum materials. Nat. Commun..

[7] Baumgartner C (2022). Supercurrent rectification and magnetochiral effects in symmetric Josephson junctions. Nat. Nanotechnol..

[8] Lyu Y-Y (2021). Superconducting diode effect via conformal-mapped nanoholes. Nat. Commun..

[9] Wakatsuki R (2017). Nonreciprocal charge transport in noncentrosymmetric superconductors. Sci. Adv..

[10] Ando F (2020). Observation of superconducting diode effect. Nature.

[11] Bauriedl L (2022). Supercurrent diode effect and magnetochiral anisotropy in few-layer NbSe_2_. Nat. Commun..

[12] Jeon K-R (2022). Zero-field polarity-reversible Josephson supercurrent diodes enabled by a proximity-magnetized Pt barrier. Nat. Mater..

[13] Pal B (2022). Josephson diode effect from cooper pair momentum in a topological semimetal. Nat. Phys..

[14] Gupta M (2023). Gate-tunable superconducting diode effect in a three-terminal Josephson device. Nat. Commun..

[15] Wu H (2022). The field-free Josephson diode in a van der Waals heterostructure. Nature.

[16] Baumgartner C (2022). Effect of Rashba and Dresselhaus spin–orbit coupling on supercurrent rectification and magnetochiral anisotropy of ballistic Josephson junctions. J. Phys. Condens. Matter.

[17] Mazur G. P. et al. The gate-tunable Josephson diode. *arXiv*10.48550/arXiv.2211.14283 (2022).

[18] Xi X (2016). Ising pairing in superconducting NbSe_2_ atomic layers. Nat. Phys..

[19] Armitage NP, Mele EJ, Vishwanath A (2018). Weyl and Dirac semimetals in three-dimensional solids. Rev. Mod. Phys..

[20] Choi Y-B (2020). Evidence of higher-order topology in multilayer WTe_2_ from Josephson coupling through anisotropic hinge states. Nat. Mater..

[21] Wu S (2018). Observation of the quantum spin Hall effect up to 100 kelvin in a monolayer crystal. Science.

[22] Kang K, Li T, Sohn E, Shan J, Mak KF (2019). Nonlinear anomalous Hall effect in few-layer WTe_2_. Nat. Mater..

[23] Xu S-Y (2018). Electrically switchable Berry curvature dipole in the monolayer topological insulator WTe_2_. Nat. Phys..

[24] Buzdin A (2008). Direct coupling between magnetism and superconducting current in the Josephson φ_0_ Junction. Phys. Rev. Lett..

[25] Konschelle F, Tokatly IV, Bergeret FS (2015). Theory of the spin-galvanic effect and the anomalous phase shift φ_0_ in superconductors and Josephson junctions with intrinsic spin-orbit coupling. Phys. Rev. B.

[26] Bergeret FS, Tokatly IV (2015). Theory of diffusive φ_0_ Josephson junctions in the presence of spin-orbit coupling. Europhys. Lett..

[27] Kononov A (2020). One-dimensional edge transport in few-layer WTe_2_. Nano Lett..

[28] Soluyanov AA (2015). Type-II Weyl semimetals. Nature.

[29] Chen C-Z (2018). Asymmetric Josephson effect in inversion symmetry breaking topological materials. Phys. Rev. B.

[30] Souto RS, Leijnse M, Schrade C (2022). Josephson diode effect in supercurrent interferometers. Phys. Rev. Lett..

[31] Qian X, Liu J, Fu L, Li J (2014). Quantum spin hall effect in two-dimensional transition metal dichalcogenides. Science.

[32] Huang C (2020). Edge superconductivity in multilayer WTe_2_ Josephson junction. Natl Sci. Rev..

[33] Li P (2017). Evidence for topological type-II Weyl semimetal WTe_2_. Nat. Commun..

[34] Kochan D, Costa A, Zhumagulov I, Žutić IJ (2023). a. e.-p. Phenomenological theory of the supercurrent diode effect: the lifshitzi Invariant. arXiv.

[35] Ilić S, Bergeret FS (2022). Theory of the supercurrent diode effect in rashba superconductors with arbitrary disorder. Phys. Rev. Lett..

[36] Ribeiro-Palau, R. et al. Twistable electronics with dynamically rotatable heterostructures. *Science*10.1126/science.aat6981 (2018).

[37] Novoselov KS, Mishchenko A, Carvalho A, Castro Neto AH (2016). 2D materials and van der Waals heterostructures. Science.

[38] Díez-Mérida J (2023). Symmetry-broken Josephson junctions and superconducting diodes in magic-angle twisted bilayer graphene. Nat. Commun..

[39] Lin J-X (2022). Zero-field superconducting diode effect in small-twist-angle trilayer graphene. Nat. Phys..

[40] Barone, A. P., G. in *Physics and Applications of the Josephson Effect* 2nd edn, 525–529 (John Wiley & Sons, 1982).

