# Supplementary material for: Intrinsic supercurrent non-reciprocity coupled to the crystal structure of a van der Waals Josephson barrier
Source: Nat Commun. 2024 Feb 6;15:1120. doi: 10.1038/s41467-024-45298-9 (PMC10847146; doi:10.1038/s41467-024-45298-9)
Supplement: Supplementary file 1 — Supplementary Information [file 41467_2024_45298_MOESM1_ESM.pdf]

Supplementary Information

Intrinsic supercurrent non-reciprocity coupled to the crystal structure of a van der Waals Josephson barrier

Jae-Keun Kim[1*†], Kun-Rok Jeon[2†], Pranava K. Sivakumar[1], Jae-Chun Jeon[1], Chris Koerner[3], Georg Woltersdorf[3] and Stuart S. P. Parkin[1*]

[1]*Max Planck Institute of Microstructure Physics, Weinberg 2, 06120 Halle (Saale), Germany*

[2]*Department of Physics, Chung-Ang University (CAU), Seoul 06974, Republic of Korea*

[3]*Department of Physics, Martin Luther University Halle-Wittenberg, Von Danckelmann Platz 3, 06120 Halle, Germany*

*To whom correspondence should be addressed: jkkim@mpi-halle.mpg.de; stuart.parkin@mpi-halle.mpg.de

[†]These authors contributed equally to this work.

S1. Optical microscopy and atomic force microscopy (AFM) images of the fabricated van der Waals WTe_2 Josephson junctions

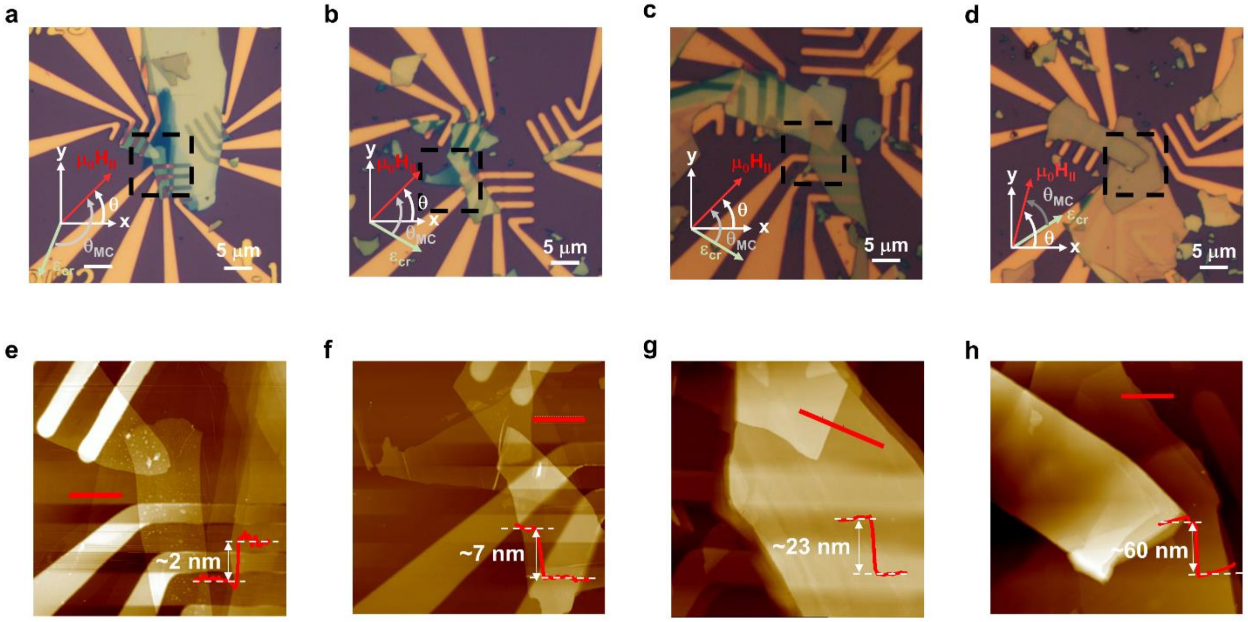

Fig. S1. In the optical microscopy images **a-d**, the direction of the internal crystal field (ε_{cr}), the relative angle between the applied field ($\mu_0 H_{\parallel}$) and ε_{cr} (θ_{MC}) and the angle of $\mu_0 H_{\parallel}$ relative to an edge of the rectangle-piece silicon wafer (θ) are shown. The black dashed box denotes the area scanned by the AFM. In the AFM images **e-h**, the scanned profile along the red line is shown as an inset in each case.

S2. Magneto-linearity and magneto-chirality of the 23-nm thick WTe_2 barrier van der Waals Josephson junction.

Figure S2a shows the $\mu_0 H_{\parallel}$-strength-dependent I_c^+ and $|I_c^-|$ of a 23-nm-thick WTe_2 barrier vdW Josephson junction (JJ). As summarized in Fig. S2b and c, the magneto-linearity and magneto-chirality, key measures of the intrinsic Josephson diode effect (see main text for details), are each confirmed for this barrier thickness.

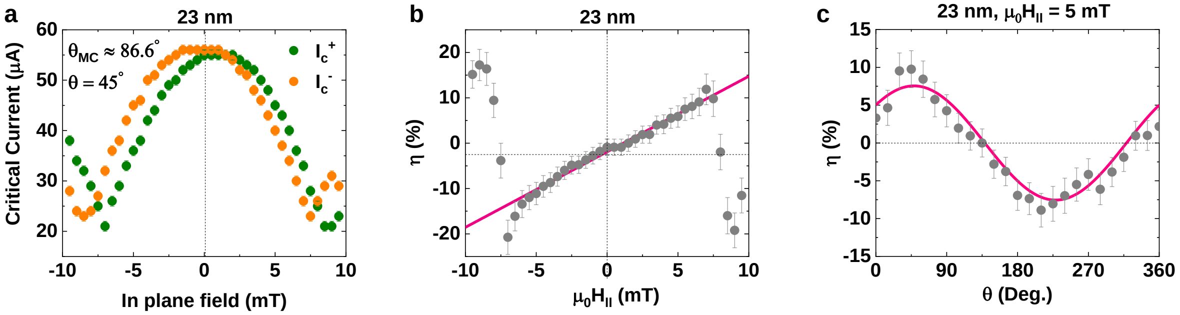

Fig. S2. a, Positive and negative Josephson critical currents $\boldsymbol{I_c^+}$ (green) and $|\boldsymbol{I_c^-}|$ (orange) *versus* in-plane (IP) magnetic field $\mu_0 H_{\parallel}$ for a $NbSe_2$/T_d-WTe_2(23 nm)/$NbSe_2$ Josephson junction. This measurement was conducted at T = 20 mK. **b,** Josephson diode efficiency ($\eta = \frac{I_c^+ - |I_c^-|}{I_c^+ + |I_c^-|}$) as a function of $\mu_0 H_{\parallel}$ and **c**, the angle dependent η of the $NbSe_2$/T_d-WTe_2(23 nm)/$NbSe_2$ junction at a fixed field, $\mu_0 H_{\parallel}$ = 5 mT.

S3. Polarization-resolved Raman measurement

Due to anisotropic lattice vibrations of WTe_2, polarization-resolved Raman spectroscopy allows us to identify the crystal orientation of the WTe_2 barriers[S1,S2]. A 532 nm laser source was used for polarization-resolved Raman measurements. Two linear polarizers were placed in front of the laser and the spectrometer, respectively. We use a corresponding definition of the angle between the incident laser light polarization and the WTe_2 flake as was used for the field-angle dependent measurement, that is θ (Fig. 3 a-c). The polarization direction is rotated by a half-wave plate. The half-wave plate was placed in front of the linear polarizer of the incident laser. In this setup, only the incident light, not the scattered light from WTe_2 flake, passes through the half-wave plate. Raman peaks at ~160 cm^{-1} and ~210 cm^{-1} were used to determine the WTe_2 crystal orientation. Fig. S 3a show these two peaks that correspond to the A1 vibrational modes of WTe_2. The Raman intensity of the A1 mode can be expressed as

$$I \propto \left|\mathrm{e}_s \cdot R(A_1) \cdot e_i^T\right|^2,$$

where $R(A_1)$ is the Raman tensor corresponding to the A_1 mode, e_i and e_s are the unit polarization vectors for the incident and scattered light, respectively, namely $\mathrm{e}_s = [0, 1, 0]$ and $\mathrm{e}_i = [\cos\theta,\ \sin\theta,\ 0]$. In this analysis the a-axis of the WTe_2 flake is along $\theta = \alpha$, so that the Raman intensity I can be written as

$$I \propto \left|\mathrm{e}_s \cdot R(A_1)' \cdot \mathrm{e}_i^T\right|^2 = \left|\mathrm{e}_s \cdot M \cdot R(A_1) \cdot M^T \cdot \mathrm{e}_i^T\right|^2 = \left|\frac{a+b}{2}\cos\theta + \frac{a-b}{2}\cos(\theta - 2\alpha)\right|^2$$

$$\text{with } R(A_1) = \begin{bmatrix} a & 0 & 0 \\ 0 & b & 0 \\ 0 & 0 & c \end{bmatrix} \text{ and } M = \begin{bmatrix} \cos\alpha & -\sin\alpha & 0 \\ \sin\alpha & \cos\alpha & 0 \\ 0 & 0 & 1 \end{bmatrix}.$$

To more accurately define the crystal orientation of WTe_2, we use the ratio of the intensity of the two A1 Raman peaks, $(I_{160cm^{-1}}/I_{230cm^{-1}})$ (26). Figure S3, b-f shows polar plots of the intensity ratio, $I_{160cm^{-1}}/I_{230cm^{-1}}$, for each thickness of the WTe_2 barrier (2, 7, 23 and 60 nm thick). The extracted values of α for each of the WTe_2 barriers correspond to 162.4°, -120.7°, 44.5° and 121.2°, respectively. Within the experimental error (a few degrees), the Raman-defined a-axis is in good agreement with that extracted from the angle dependent diode data (Fig. 3 a-c).

Thus, the polarization-resolved Raman spectroscopy strongly supports a crystal-structure-driven magneto-chiral Josephson diode effect in our vdW vertical JJs.

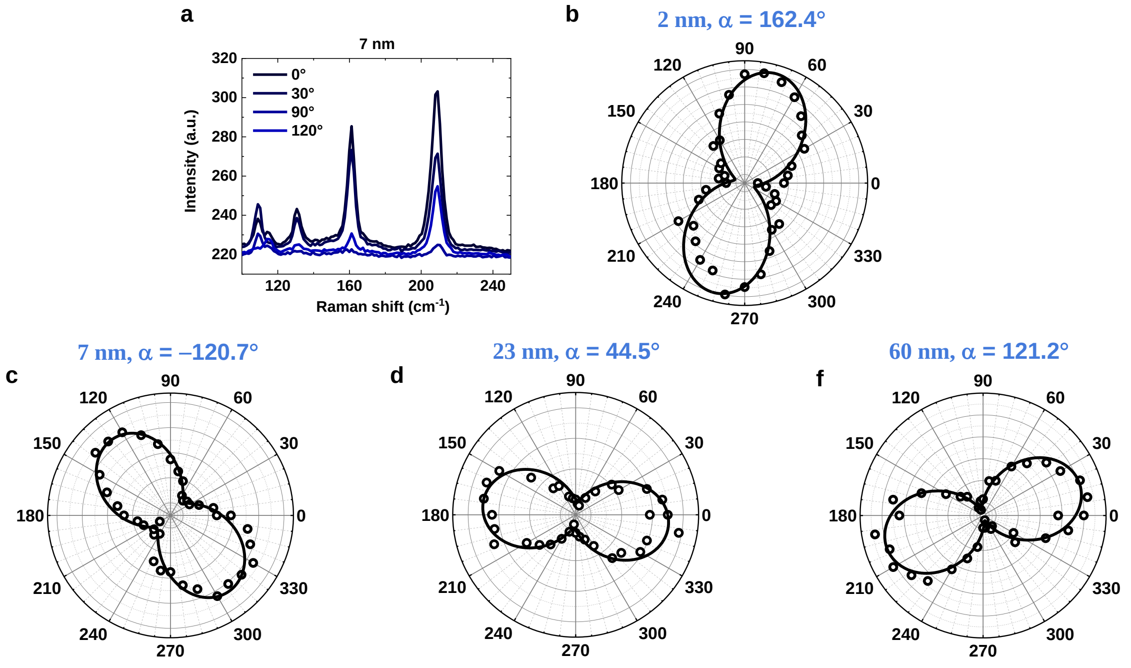

Fig. S3. Polarization-resolved Raman spectra for WTe_2. a, Representative Raman spectra for four selected polarization angles of a 7-nm-thick WTe_2 flake **b-f,** Polar plot of the polarization angle dependent intensity ratio $I_{160cm^{-1}}/I_{230cm^{-1}}$ for 2, 7, 23 and 60 nm thick WTe_2 flakes, respectively. The extracted rotation angle of each WTe_2 flake is noted above each polar plot.

S4. Control vdW Josephson junction with a $MoTe_2$ barrier

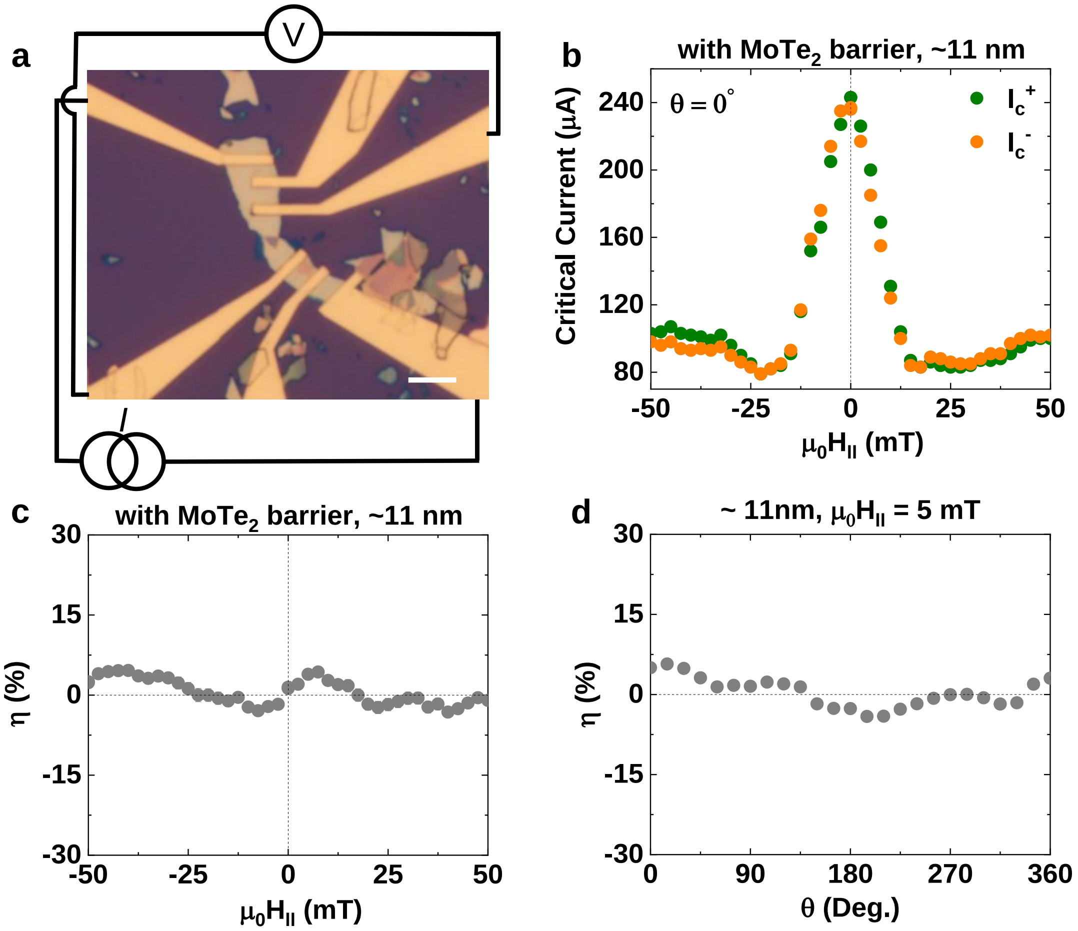

Fig. S4. Suppression of magneto-linearity and magneto-chirality in supercurrents through a locally centrosymmeric vdW Josephson barrier a, Optical micrograph of $MoTe_2$ Josephson junctions. Scale bar is 5 μm. Note that unlike the orthorhombic phase T_d-WTe_2[S1,S2,S3], the monoclinic phase 1*T*′-$MoTe_2$ is centrosymmetric[S4]. Our $MoTe_2$ flake (with around 11 nm) may possess a *mixed phase* of T_d and 1*T*′ at low temperatures as recently discussed in detail in [S5,S6]. **b,** Positive and negative Josephson critical current I_c^+ (green) and $|I_c^-|$ (orange) *versus* in-plane magnetic field $\mu_0 H_{\parallel}$ for a 11 nm thick $MoTe_2$ barrier in a $NbSe_2/MoTe_2/NbSe_2$ Josephson junction. The measurement was conducted at $T = 2$ K. Josephson diode efficiency ($\eta = \frac{I_c^+ - |I_c^-|}{I_c^+ + |I_c^-|}$) as a function of magnetic field strength (**c**) and angle (**d**) for the $NbSe_2/MoTe_2/NbSe_2$ Josephson junction.

S5. Decay length of Josephson supercurrents

For *short ballistic* JJs ($d < \xi < l_{\mathrm{mfp}}$), the critical supercurrent (I_c) is described as $I_c = N\frac{2\pi e\Delta}{h}$. Here N is the number of transverse modes at the Fermi energy level, through which supercurrents can propagate, Δ is the superconducting energy gap, h is the Planck constant, d is the junction length and ξ is the junction's coherence length[S5]. As the zero-bias normal-state resistance (R_N) of the ballistic JJs is given by $R_N = \frac{h}{2e^2N}$, one can expect the characteristic voltage ($V_C = I_C R_N = \frac{\pi\Delta}{e}$) in the short junction limit ($d < \xi$) to be independent of d. On the other hand, for *long ballistic* JJs ($\xi < d < l_{\mathrm{mfp}}$), V_C is expected to scale inversely with d due to the decreased Thouless energy of the junctions, $V_C \propto 1/d$[S7]. In a prior study, lateral $Nb/WTe_2/Nb$ JJs[S8], where transparent/direct contacts were formed across the Nb/WTe_2 and WTe_2/Nb interfaces, revealed the expected $1/d$ dependence in the $V_C(d)$ data when reaching the long junction limit ($\xi < d$).

However, our vertical $NbSe_2/WTe_2/NbSe_2$ vdW JJs have distinct $V_C(d)$ properties. V_C decreases exponentially with increasing the WTe_2 thickness, $V_C \propto \exp\left(-\frac{d}{\xi}\right)$, as displayed in Fig. S5. This result can be explained by considering the vdW gap formed at either side of the WTe_2 Josephson barrier which is expected to act as a tunnel barrier at the interface between $NbSe_2$ and WTe_2 flakes.

To quantify the *effective* ξ value of Josephson supercurrent through our vdW gap/WTe_2/vdW gap *composite* barrier, we fit the WTe_2 thickness (d) dependent V_C with an exponential decay function, $\exp\left(-\frac{d}{\xi}\right)$, to obtain $\xi = 31.6$ nm (Fig. S5).

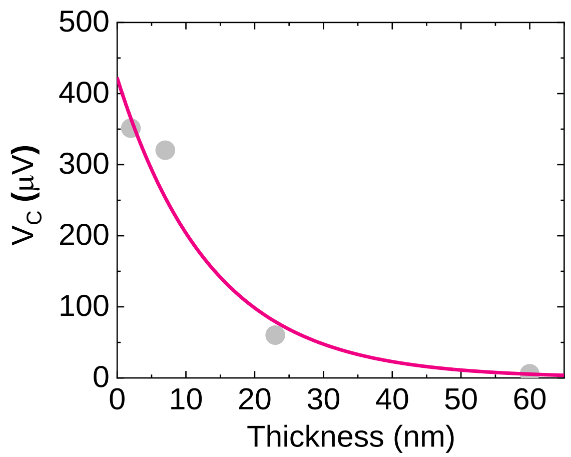

Fig. S5. Estimation of the coherence length. Characteristic voltage ($V_C(d) = I_C R_N(d)$) as a function of WTe_2 thickness (d) measured at T = 20 mK. The pink line indicates an exponential fit.

S6. Reference vdW Josephson junction with *no* WTe_2 barrier

Direct tunneling between two adjacent superconducting leads may play a role in the transport properties of JJs, in particular, for those with atomically thin barriers. In the case of the 2-nm-thick WTe_2 barrier, we expect two different charge-transport mechanisms, 1) direct tunneling (of charge) from one to the other superconducting lead, and 2) ballistic transport[S9] through Andreev bound states (ABS) formed in the WTe_2 barrier. Given that the 2-nm-thick WTe_2 barrier junction clearly reveals magneto-chiral characteristics with reference to its polar axis, one can conclude the latter ballistic transport dominates over the former direct tunneling, which is expected to be independent of the crystal structure of WTe_2.

Nevertheless, for the sake of completeness, we have fabricated WTe_2-absent $NbSe_2$/$NbSe_2$ JJs, where the direct tunneling of charge between the superconducting electrodes is solely responsible for the Josephson transport. Recall that the vdW gap formed in-between top and bottom $NbSe_2$ flakes can serve as a tunnel barrier, making up $NbSe_2$/vdW gap/$NbSe_2$ vertical JJs[S10]. None of the magneto-linearity and the magneto-chirality effects are detected in these WTe_2-absent JJs (Fig. R3), as would be expected for the vdW gap acting as a *crystal-structure-less* Josephson barrier.

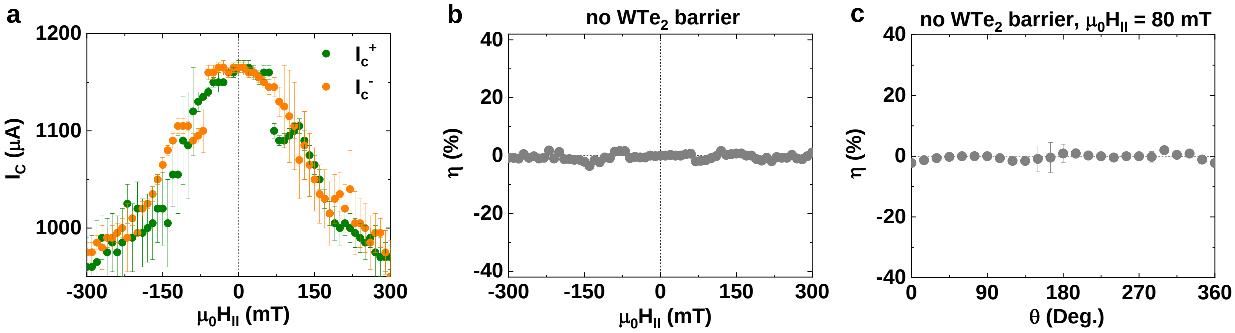

Fig. S6. Absence of magneto-linearity and magneto-chirality in supercurrents of the WTe_2-absent $NbSe_2$/$NbSe_2$ JJ. a, Positive and negative Josephson critical current I_c^+ (green) and $|I_c^-|$ (orange) *versus* in-plane magnetic field $\mu_0 H_{\parallel}$ for the WTe_2-absent JJ. All measurements were conducted at T = 2 K. Josephson diode efficiency $\eta (= \frac{I_c^+ - |I_c^-|}{I_c^+ + |I_c^-|})$ as a function of magnetic field strength (**b**) and angle (**c**) for the WTe_2-absent JJ. We note that the diode efficiency (η) < few % for the WTe_2-absent JJ is remarkably small compared with the WTe_2-present JJs and none of the magneto-linearity and the magneto-chirality are evident in the WTe_2-absent JJ.

S7. Instrument limitation for measuring Josephson junctions with high critical currents.

At the base temperature (of our measurement setup), a heating effect is unavoidable if the applied dc current to JJs goes beyond a few hundred μA. To avoid this unintentional heating effect, we had no choice but to measure 2 nm and 7 nm thick barrier JJs at 200 mK.

Nonetheless, the *I*-*V* curves of 7 nm thick barrier JJs reveal no significant difference between 200 and 20 mK (Fig. S7), as would be expected for the measurement temperature of ≤ $0.3T/T_c$. This explains why the critical currents of 2 nm (a) and 7 nm (b) thick barrier JJs measured at 200 mT are still much higher than those of 60 nm (c) thick barrier junction at 20 mK.

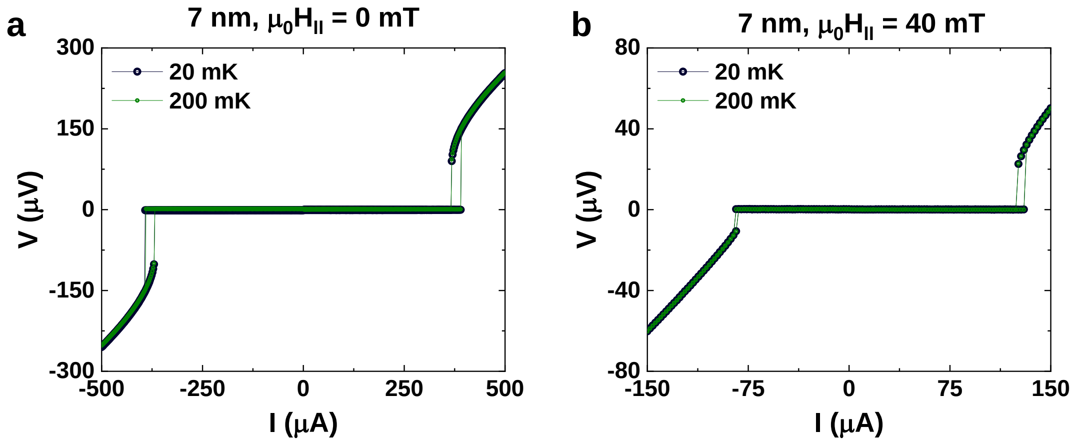

Fig. S7. Current-Voltage *I*-*V* curves of a van der Waals JJ with 7 nm thick T_d-WTe_2 barrier measured at T = 20 (black) , 200 mK (green) with **(a)** $\mu_0 H_{\|}$ = 0 mT and **(b)** $\mu_0 H_{\|}$ = 40 mT.

S8. Kinetic inductance effect of superconducting electrodes

The kinetic inductance $L_K \approx \mu_0 \frac{\lambda^2 l}{wt}$ of thin $NbSe_2$ flakes in WTe_2 JJs is estimated to be ~ 33 pH for zero current and at zero temperature (where L_K is the smallest). Here, μ_0 is vacuum permeability and λ is the London penetration depth of bulk $NbSe_2$ (230 nm)[S11]. *l*, *w* and *t* are the length, width and thickness of $NbSe_2$ electrodes, respectively. The estimated L_K value of our junctions is at least one order of magnitude smaller than that in a previous study[S12]. Furthermore, our measurement setup under application of in-plane magnetic fields differs from their one where supercurrent non-reciprocity appears even in the absence of magnetic fields (i.e. no need for breaking the time-reversal symmetry). As a result, we can conclude that the kinetic inductance contribution to the supercurrent non-reciprocity of our WTe_2 JJs is insignificant.

We emphasize that the $\mu_0 H_{\parallel}$ angular dependence of η of WTe_2 Josephson junctions is intimately coupled to the crystal orientation of each WTe_2 barrier. So, we do not believe that the kinetic inductance (of extrinsic effects) is responsible for the magneto-chirality of WTe_2 JJs. In addition, the absence magneto-chirality in control devices (WTe_2-absent and *1T′*-$MoTe_2$ barrier JJs) decisively supports our intrinsic picture.

S9. Influence of a possible small vertical magnetic field due to a misalignment

Although there exists a possibility for the existence of a non-zero vertical component of applied in-plane magnetic fields due to few-degrees misalignment, it cannot explain the crystal-orientation- coupled supercurrent non-reciprocity of our WTe_2 JJs. This is because the non-zero vertical (misalignment) field is expected to *be independent of* rotating the in-plane magnetic field with reference to the polar axis of WTe_2. Thus, any small vertical misalignment cannot explain our data. We would like to re-emphasize that in 4 distinct junctions where we carried the WTe_2 thickness we demonstrated clearly from polarized Raman studies after we had carried out our studies of the dependence of the η on the in-plane field angle that η was clearly associated with the polar direction within the WTe_2 plane. In these 4 cases the orientation of this polar direction was different with respect to the device contact geometry. Since any vertical misalignment would likely be the same for all junctions this rules out any possible influence of any such misalignment. Thus we have clearly shown the intrinsic magneto-chiral origin of the Josephson diode effect. In addition, the absence of the magneto-chirality of $MoTe_2$ and WTe_2-absent control devices allows one to further exclude the possibility of the non-zero vertical (misalignment) field.

References

S1. Choi, Y.-B. *et al.* Evidence of higher-order topology in multilayer WTe_2 from Josephson coupling through anisotropic hinge states. *Nat. Mater.* **19**, 974-979 (2020).

S2. Kang, K., Li, T., Sohn, E., Shan, J. & Mak, K. F. Nonlinear anomalous Hall effect in few-layer WTe_2. *Nat. Mater.* **18**, 324-328 (2019).

S3. Xu, S.-Y. *et al.* Electrically switchable Berry curvature dipole in the monolayer topological insulator WTe_2. *Nat. Phys.* **14**, 900-906 (2018).

S4. Qian, X., Liu, J., Fu, L. & Li, J. Quantum spin Hall effect in two-dimensional transition metal dichalcogenides. *Science* **346**, 1344-1347 (2014).

S5. Paul, S. *et al.* Tailoring the phase transition and electron-phonon coupling in 1T′-$MoTe_2$ by charge doping: A Raman study. Phys. Rev. B **102**, 054103 (2020).

S6. Cheon Y. *et al.* Structural Phase Transition and Interlayer Coupling in Few-Layer 1T′ and T_d $MoTe_2$. ACS Nano **15**, 2962-2970 (2021).

S7. Beenakker, C. W. J. Three “Universal” Mesoscopic Josephson Effects. In: Transport Phenomena in Mesoscopic Systems, edited by H. Fukuyama and T. Ando (Springer, Berlin, 1992).

S8. Huang, C. *et al.* Edge superconductivity in multilayer WTe_2 Josephson junction, *Natl. Sci. Rev.* **7**, 1468–1475 (2020).

S9. Baumgartner, C. *et al.* Supercurrent rectification and magnetochiral effects in symmetric Josephson junctions. *Nat. Nanotechnol.* **17**, 39-44 (2022).

S10. Yabuki, N. *et al.* Supercurrent in van der Waals Josephson junction. *Nat. Commun.* **7**, 10616 (2016).

S11. Trey, P., Gygax, S. & Jan, J.-P. Anisotropy of the Ginzburg-Landau parameter κ in $NbSe_2$. *J. Low Temp. Phys.* **11**, 421 (1973)

S12. Schönle *et al.* Field-Tunable 0-π-Transitions in SnTe Topological Crystalline Insulator SQUIDs. Sci. Rep. **9**, 1987 (2019)
